# Effect of voluntary waiting period on metabolism of dairy cows during different phases of the lactation

**DOI:** 10.1093/jas/skad194

**Published:** 2023-06-09

**Authors:** Eline E A Burgers, Roselinde M A Goselink, Rupert M Bruckmaier, Josef J Gross, Ruurd Jorritsma, Bas Kemp, Akke Kok, Ariette TM van Knegsel

**Affiliations:** Adaptation Physiology Group, Wageningen University and Research, NL-6700 AH Wageningen, the Netherlands; Wageningen Livestock Research, Wageningen University and Research, NL-6700 AH Wageningen, the Netherlands; Wageningen Livestock Research, Wageningen University and Research, NL-6700 AH Wageningen, the Netherlands; Veterinary Physiology, Vetsuisse Faculty, University of Bern, CH-3012 Bern, Switzerland; Veterinary Physiology, Vetsuisse Faculty, University of Bern, CH-3012 Bern, Switzerland; Department of Farm Animal Health, Ruminant Health Unit, Utrecht University, NL-3508 TD Utrecht, the Netherlands; Adaptation Physiology Group, Wageningen University and Research, NL-6700 AH Wageningen, the Netherlands; Adaptation Physiology Group, Wageningen University and Research, NL-6700 AH Wageningen, the Netherlands; Adaptation Physiology Group, Wageningen University and Research, NL-6700 AH Wageningen, the Netherlands

**Keywords:** energy partitioning, extended calving interval, extended lactation, individual cow variation, metabolic status

## Abstract

An extended calving interval (CInt) by extending the voluntary waiting period (VWP) could be associated with altered metabolism in dairy cows. The aim of this study was first to evaluate the effects of VWP on metabolism and body condition during the first 305 d after the first calving in the experiment (calving 1), around the end of the VWP, and during pregnancy (280 d before calving 2). Second, the effects of the VWP on metabolism were determined from 2 wk before until 6 wk after calving 2. Third, individual cow characteristics were used to predict milk production and body condition of cows after different VWP. Holstein-Friesian cows (*N* = 154, 41 primiparous [PP], 113 multiparous [MP]) were blocked for parity, milk production, and lactation persistency, randomly assigned to a VWP of 50, 125, or 200 d (VWP50, VWP125, or VWP200) and followed from calving 1 until 6 wk after calving 2. In the first 6 wk after calving 1 and from 2 wk before until 6 wk after calving 2, weekly plasma samples were analyzed for nonesterified fatty acids (NEFA), β-hydroxybutyrate, glucose, insulin, and insulin-like growth factor 1 (IGF-1). From wk 7 after calving 1 until 2 wk before calving 2, insulin and IGF-1 were analyzed every 2 wk. Fat- and protein-corrected milk (FPCM) and body weight (BW) gain were measured weekly. Cows were divided in two parity classes based on calving 1 (PP and MP) and remained in these classes after calving 2. During pregnancy, MP cows in VWP200 had greater plasma insulin and IGF-1 concentration and lower FPCM compared with MP cows in VWP125 (insulin: 18.5 vs. 13.9 µU/mL, CI 13.0–19.7, *P* < 0.01; IGF-1: 198.5 vs. 175.3 ng/mL ± 5.3, *P* = 0.04; FPCM: 22.6 vs. 30.0 kg/d ± 0.8, *P* < 0.01) or VWP50 (insulin: 15.8 µU/mL, *P* < 0.01; IGF-1: 178.2 ng/mL, *P* < 0.01; FPCM: 26.6 kg/d, *P* < 0.01) and had a greater daily BW gain compared with cows in VWP50 (3.6 vs. 2.5 kg/d ± 0.2; *P* < 0.01). After calving 2, MP cows in VWP200 had greater plasma NEFA concentration (0.41 mmol/liter) compared with MP cows in VWP125 (0.30 mmol/liter, *P* = 0.04) or VWP50 (0.26 mmol/liter, *P* < 0.01). For PP cows, the VWP did not affect FPCM or body condition during the first lactation in the experiment, or metabolism after calving 2. Independent of the VWP, higher milk production and lower body condition before insemination were associated with higher milk production and lower body condition at the end of the lactation. Variation in these characteristics among cows could call for an individual approach for an extended VWP.

## Teaser

An extended voluntary waiting period (VWP) reduces the frequency of calvings, but for multiparous cows, it increases the risk for fattening at the end of the lactation. An individually adapted VWP may reduce this risk.

## Introduction

Extending the calving interval (CInt) by extending the voluntary waiting period (VWP) is a strategy to reduce the frequency of calvings. This strategy could thereby reduce the risk for diseases per unit of time, as most diseases are associated with the period around calving (Friggens et al., 2004). An extended VWP could be associated with altered metabolic status and energy partitioning in dairy cows during different phases in the lactation. First, insemination takes place later in lactation, when milk production is decreased (Gaillard et al., 2016). Consequently, insulin and insulin-like growth factor 1 (IGF-1) may be increased (Lucy, 2008), and reproductive performance may be improved (Wathes et al., 2007; Niozas et al., 2019a). Second, when the VWP and thus the lactation was extended, cows had more persistent lactation curves (Niozas et al., 2019b; Burgers et al., 2021a). Third, when the VWP was extended, cows had a lower milk production and increased body condition score (BCS) at the end of lactation (Niozas et al., 2019b; Burgers et al., 2021a). These differences in persistency, milk production, and body condition indicate a difference in energy partitioning between milk and body reserves and could be related to changes in hormone concentrations during lactation. During mid and late lactation, cows may be in a more positive energy balance (EB). As a result, plasma glucose is higher and insulin secretion is stimulated (Hove, 1978; Holtenius and Holtenius, 1996; Zinicola and Bicalho, 2019). Furthermore, a greater plasma insulin concentration was related to more energy partitioning toward body reserves and less toward milk (Hart, 1983; Lucy, 2008).

Additionally, cows with an extended VWP have more risk for an increased body condition at the end of the lactation (Niozas et al., 2019b; Burgers et al., 2021a). As a result, they could have a reduced feed intake and a more negative EB after the next calving. A more negative EB can be expected to increase the plasma concentration of nonesterified fatty acids (NEFA) and β-hydroxybutyrate (BHB), and herewith cows with an extended VWP in the previous lactation might have an increased risk for metabolic disorders after calving (Gillund et al., 2001; Roche and Berry, 2006).

Not all cows with an extended VWP, however, have a reduced milk yield or an increased body condition at the end of the lactation, which could indicate that some cows might be more prone to partition energy toward milk rather than toward body reserves (Lehmann et al., 2016; Niozas et al., 2019b). For example, cows with a high milk production at 450 days in milk (DIM; 18.9 kg/d) had a tendency for more lipid mobilization around 460 DIM and around 580 DIM compared with cows with a lower milk production at 450 DIM (12.3 kg/d; Marett et al., 2019). In addition, individual cow characteristics, such as milk production and body weight (BW) in early lactation, were associated with milk production per day of CInt and lactation persistency in extended lactations (Burgers et al., 2021a). It can be hypothesized that milk production and fattening at the end of the lactation are related with milk production and body condition in early lactation, but also with insulin and IGF-1 concentration. Early identification of cows that are able to maintain milk production in an extended lactation is beneficial for selection of cows for an extended VWP.

The aim of this study was first to evaluate the effects of 3 VWP (50, 125, and 200 d) on insulin, IGF-1, and body condition during different phases of the lactation: during the first 305 d after the first calving in the experiment (calving 1), around the end of the VWP, and during the pregnancy period (i.e., period of 280 d before calving 2). Second, the effects of the VWP and CInt on metabolic status from 2 wk before until 6 wk after calving 2 were determined by analyzing body condition, dry matter intake (DMI), EB, plasma NEFA, BHB, glucose, insulin, and IGF-1 concentration. Third, individual cow characteristics, as monitored before successful insemination, were used to predict three variables: 1) Mean FPCM during the final 6 wk before dry-off, 2) Mean BCS during the final 12 wk before dry-off, and 3) FPCM per day of CInt. These three variables are important for a successful extended lactation to predict the risk for individual cows for a low FPCM end lactation, a high BCS end lactation, and a low FPCM per day of CInt.

## Materials and Methods

### Animals and housing

The experimental protocol was approved by the Institutional Animal Care and Use Committee of Wageningen University and Research (the Netherlands) and complied with the Dutch law on Animal Experimentation (protocol number 2016.D-0038.005). The experiment was conducted at Dairy Campus research farm (Leeuwarden, the Netherlands) between December 2017 and January 2020.

This publication is part of a large and long-time study that has multiple companion papers addressing extended lactations in relation with milk yield (Burgers et al., 2021a), economic performance (Burgers et al., 2022), fertility (Ma et al., 2022a), and udder health (Ma et al., 2022b). In the current publication, body condition, hormones, and metabolites are presented during different phases of the extended lactation and feed intake, EB, and hormones and metabolites are presented during the start of the subsequent lactation. The animals, experimental design, and treatments have been described earlier (Burgers et al., 2021a). In short, 154 cows were selected from a research herd of 500 lactating Holstein Friesian cows based on the following criteria: no twin pregnancy, no clinical mastitis or somatic cell count (SCC) > 250,000 at the final two milk test days before dry-off, and expected to finish a complete lactation based on being in good general health. For the current study, cows were followed from 2 wk before expected calving (calving 1) until 6 wk after calving 2 or until culling. Cows were housed in a freestall barn and milked twice daily around 0600 and 0600 hours in a 40-cow rotary milking parlor (GEA, Dusseldorf, Germany). During lactation, cows were fed with partially mixed ration (PMR): grass silage, corn silage, soybean meal, and wheat meal, supporting 22 kg of milk. Moreover, concentrate was supplied from the day of calving and increased until 21 DIM to 9 kg/d (for primiparous [PP] cows) or 10 kg/d (for multiparous [MP] cows). After 100 DIM, concentrate supply was decreased based on reductions in milk production. In the milking parlor, cows additionally received 1 kg of concentrate per day. Ration during the dry period consisted of grass silage and corn silage, supplemented with wheat straw and concentrate. In the last 10 d before the expected calving date, cows received 1 kg concentrate daily. Once per week, cows between 42 and 49 d before the expected calving date were dried-off. In the 7 d prior to dry-off, cows were given the dry-cow ration. During the last 3 d before dry-off, cows were milked once daily. When cows had an SCC > 150,000 cells/mL at the final milk test day, cows were treated with antibiotics at dry-off (Orbenin Dry Cow Extra, Zoetis, the Netherlands). All cows were treated with teat sealant at dry-off (Orbeseal, Zoetis, the Netherlands).

### Experimental design

**Figure 1.**
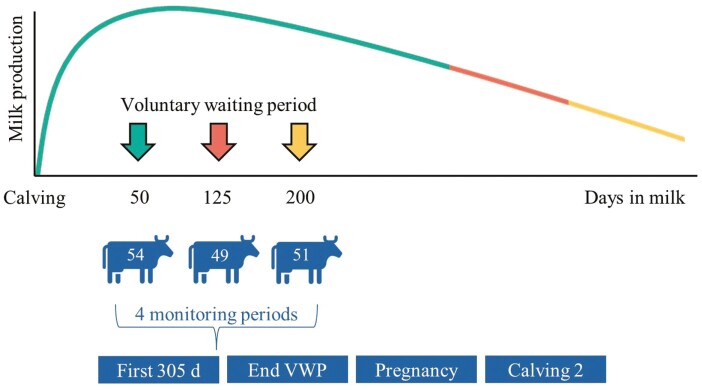
Experimental design showing the three VWPs with their moment of start of insemination, the number of cows per VWP, and the monitoring periods within the study.

Figure 1 shows an overview of the experimental design and the different monitoring periods. The selected 154 animals (41 PP, 113 MP) were blocked for parity, calving date, milk production in the previous lactation (MP cows) or expected milk production (PP cows), and the breeding value for persistency (CRV, Arnhem, the Netherlands) in week 6 after calving. Each block consisted of three cows. Per block, the cows were randomly divided over three treatment groups: a VWP of 50 d (**VWP50**), 125 d (**VWP125**), or 200 d(**VWP200**). Cows in the three treatment groups were inseminated at the first estrus after the end of their VWP. Estrus detection was carried out by using neck mounted 3D accelerometers (Nedap Smarttag Neck, Groenlo, the Netherlands) in combination with visual observations by the animal caretaker. Cows were inseminated until 300 DIM. Cows that did not conceive within 300 DIM stayed in the experiment until 530 DIM as long as they produced at least 10 liters of milk/d.

### Measurements and calculations

#### Milk production and body condition.

Milk production was recorded at every milking. Milk samples were collected for each individual cow 4 times/wk (Tuesday afternoon, Wednesday morning, Wednesday afternoon, Thursday morning) in 10 mL tubes containing Bronopol as a preservative and analyzed for the percentage of fat, protein, and lactose as a pooled sample ([ISO 9622, 2013]; Qlip, Zutphen, the Netherlands). The FPCM was calculated as follows (CVB, 2016):

FPCM (kg) = milk (kg) × (0.337 + 0.116 × fat (%) + 0.06 × protein (%)),

by using the weekly contents of fat and protein and the mean daily milk production of each week. BCS was visually evaluated every 4 wk by the same person using a 1 to 5 scale (Ferguson et al., 1994). BW was measured twice daily after milking, by a scale that the cows walked over when returning from the milking rotary to the freestall (GEA). Dry cows were weighed once a week on the same scale. The difference in BW between two subsequent wk (ΔBW) was calculated by subtracting average BW in 1 wk from average BW in the next wk. The ΔBW was only calculated if BW was recorded at least five times in both weeks.

#### Energy balance and dry matter intake.

Weekly EB was calculated in the first 6 wk after calving 1, and from 2 wk before until 6 wk after calving 2, as the difference between intake of NE and requirements of NE for maintenance, milk production, and pregnancy (CVB, 2016). To calculate intake of NE, daily intake of PMR was measured individually by using roughage intake control (RIC) troughs (Hokofarm, Emmeloord, the Netherlands) with at least 1 trough available per 2 cows. Concentrate intake was recorded individually for all cows, by using concentrate feeders (Hanskamp, Doetinchem, the Netherlands) that measure intake per cow. To calculate intake, the rest concentrate was subtracted from the concentrate that was offered. The NE requirement for maintenance was assumed to be 291.18 kJ/BW^0.75^, NE requirement for milk production was assumed to be 3,049.8 kJ/kg FPCM, and NE requirement for pregnancy was assumed to be 18,630 kJ/d in the final 2 wk before calving (CVB, 2016). Energy intake, requirements, and EB are expressed in kJ/BW^0.75^ per day. DMI of PMR and concentrate was calculated separately and added together to calculate total DMI. The PMR: concentrate ratio was calculated by dividing the DMI of PMR over the DMI of concentrate.

#### Blood collection and analysis.

Blood was collected weekly in the first 6 wk after calving 1 in the experiment, and from 2 wk before until 6 wk after calving 2, for the analysis of plasma NEFA, BHB, glucose, insulin, and IGF-1 concentration. From 7 wk after calving 1 until 2 wk before calving 2, blood was collected every 2 wk for the analysis of plasma insulin and IGF-1 concentration. After the morning milking, blood (10 mL) was collected from the coccygeal vessels into evacuated EDTA tubes (Vacuette, Greiner BioOne, Kremsmunster, Austria). Blood samples were kept on ice before centrifugation for plasma isolation (3,000 × *g* for 15 min, 4 °C). Samples were stored at −20 °C. Plasma insulin concentration was measured by using kit no. PI-12K from EMD Millipore Corporation (Billerica, MA, USA). Plasma IGF-1 concentration was measured by using kit no. A15729 from Beckman Coulter (Fullerton, CA, USA). Plasma metabolite concentration was measured by using an autoanalyzer (Cobas Mira, Roche), with the following enzymatic kits: NEFA: NEFA FA115; BHB: RANBUT RB1007; Glucose: GLUC-PAP GL364 (Randox Laboratories Ltd., Schwyz, Switzerland). The inter- and intra-assay CV for insulin and IGF-1 was <15%. The inter- and intra-assay CV for NEFA, BHB, and glucose was <1%.

### Individual cow characteristics

Individual cow characteristics were used to predict three variables: mean FPCM in the final 6 wk before dry-off, mean BCS in the final 12 wk before dry-off, and FPCM per day of CInt. Available individual cow characteristics used in the prediction models consisted of milk production in the previous lactation (MP cows) or expected milk production (PP cows), the breeding value for persistency (CRV), and cow characteristics in three periods: 1) the first 6 wk of lactation, 2) between calving and successful insemination, and 3) the final week before successful insemination. Available characteristics in the first 6 wk of lactation were: EB, DMI of concentrate and PMR, and plasma NEFA, BHB, and glucose concentration. Available characteristics between calving and successful insemination were peak production, day of peak production, slope to peak production, slope from peak production until day of successful insemination, and slope in the 3 wk before successful insemination. Peak production was calculated as the greatest 5-d rolling average milk production between calving and successful insemination. Slope to peak production was calculated as the slope in kg milk per day from day 10 in lactation until day of peak production and was computed as peak production minus the 5 d rolling average of the milk production on day 10 in lactation (i.e., the average milk production from day 8 until day 12), divided by the day of peak production minus 10. Slope from peak production until successful insemination was calculated the same way, using the peak production and the 5 d rolling average of the final 5 d before successful insemination. Slope in the last 3 wk before successful insemination was calculated the same way. Available characteristics before successful insemination were mean milk production, mean fat, protein, and lactose content in the milk, fat to protein ratio, mean BW, mean BCS (final month before successful insemination), mean SCC, and mean plasma insulin and IGF-1 concentration.

### Statistical analysis

One cow in VWP 50 was culled on day 43 after calving, therefore 153 cows were included in the analysis (41 PP, 112 MP). Parity (PP or MP) refers to the parity of the cow after the first calving in the experiment. Statistical analyses were performed by using SAS version 9.4 (SAS Institute Inc., Cary, NC). Normality was assessed by visual inspection. For all models, plasma NEFA, BHB, and glucose concentration, plasma insulin concentration, SCC, slope to maximum production, and slope from maximum production to successful insemination were transformed to their natural logarithm to approximate a normal distribution. In the model for the 6 wk after calving 2 in the experiment, DMI of concentrate was transformed to the 10th power to approximate a normal distribution. Values are presented as least-squares mean (LSM) ± SEM. All *P*-values of pair-wise comparisons of LSM were corrected with the Bonferroni-adjustment according to the number of comparisons per analysis.

First, data were analyzed separately for three periods during different phases of the lactation: first 305 d after calving 1, 8 wk around the end of the VWP (i.e., around the start of the insemination period; day −28 to 28 relative to the end of the VWP), and the pregnancy period (i.e., period of 280 d before calving 2). Second, data were analyzed from 2 wk before until 6 wk after calving 2. Third, individual cow characteristics as monitored before successful insemination were used to predict mean FPCM in the final 6 wk before dry-off, mean BCS in the final 12 wk before dry-off, and FPCM per day of CInt of cows after different VWP. The first 6 wk after calving 1 in the experiment were not analyzed separately as no effect of VWP can be apparent during that time; this data was only used in the prediction models.

#### First 305 d after calving 1.

During the first 305 d after calving 1, plasma insulin and IGF-1 concentration, BCS, BW, and ΔBW were analyzed for fixed effects of VWP (VWP50, VWP125, or VWP200), parity (PP or MP), lactation week (weeks 1 to 44), and their two-way interactions. A repeated measurements model (PROC MIXED) was used for this analysis, with a repeated effect of lactation week with cow as the repeated subject. Plasma insulin and IGF-1 concentrations were measured once every 2 wk between 7 and 44 wk in lactation. The analyses were done for all cows (*N* = 153), and for cows that had a second calf in the experiment (*N* = 127).

#### Eight week around the end of the VWP.

During the 8 wk around the end of the VWP, plasma insulin and IGF-1 concentration, BCS, BW, and ΔBW were analyzed for fixed effects of VWP (VWP50, VWP125, or VWP200), parity (PP or MP), week relative to the end of the VWP (−3, −1, 1, 3; where days −28 to −14 is week −3; days −14 to −1 is week −1; days 1 to 14 is week 1; and days 14 to 28 is week 3), and their two-way interactions. A repeated measurements model (PROC MIXED) was used for this analysis, with a repeated effect of lactation week with cow as the repeated subject. The analyses were done for all cows that reached the end of the VWP (*N* = 151), and for cows that had a second calf in the experiment (*N* = 127).

#### Pregnancy period.

During the pregnancy period, plasma insulin and IGF-1 concentration were analyzed for fixed effects of VWP, parity (PP or MP), week relative to calving 2 (weeks −40 to −1), and their two-way interactions. A repeated measurements model (PROC MIXED) was used for this analysis, with a repeated effect of week to calving with cow as the repeated subject. The analysis was done for all cows that had a second calf in the experiment (*N* = 127). For the final 34 wk before dry-off (40 wk pregnancy minus 6 wk dry period), BCS, BW, and ΔBW were analyzed for fixed effects of VWP, parity, week relative to dry-off (weeks −34 to −1), and their two-way interactions. A repeated measurements model (PROC MIXED) was used for this analysis, with a repeated effect of week to dry-off with cow as the repeated subject. The analysis was done for all cows that had a second calf and a dry period in the experiment (*N* = 124).

#### Two week before until six week after calving 2.

During the period around calving 2, BCS, BW, DMI, EB, and plasma NEFA, BHB, glucose, insulin and IGF-1 concentration were analyzed for fixed effects of VWP (VWP50, VWP125, or VWP200), parity (PP or MP, referring to the parity of the cow in the first lactation in the experiment), week relative to calving 2 (week −2 to 6), and their two-way interactions. A repeated measurements model (PROC MIXED) was used for this analysis, with a repeated effect of lactation week with cow as the repeated subject. The periods before calving and after calving were analyzed separately. The analysis was done for all cows that had a second calf in the experiment (*N* = 127).

#### *Individual cow characteristics*.

Cow characteristics between calving 1 and successful insemination were evaluated as a predictor for mean FPCM in the final 6 wk before dry-off, mean BCS in the final 12 wk before dry-off, and FPCM per day of CInt of cows after different VWP. Evaluations were based on data of cows that had a second calf and a dry period in the experiment (*N* = 124). First, the effect of VWP, parity, and their interaction on the three variables was tested with a general linear model in SAS (PROC MIXED). Second, the effect of each cow characteristic on the three variables was tested in a univariate analysis, but always including the effect of parity, with a general linear model in SAS (PROC MIXED). The VWP was not included in the prediction models, to predict milk production and body condition based on cow characteristics before successful insemination independent of the VWP. Third, when *P*-value was <0.20 (Van Hoeij et al., 2016), the cow characteristic was included in the multivariable model. The multivariable model always included parity as fixed effect. The cow characteristics and their interaction with parity stayed in the model if *P* < 0.05 by using backward selection. Finally, the adjusted R^2^ of the final multivariable model was compared with the adjusted R^2^ of the model with only VWP, parity, and their interaction when significant, to investigate added value of the cow characteristics in the model instead of VWP to predict the three variables. Adjusted R^2^ was calculated as follows:


$$\text{Adjusted }{\text{R}}^{2}=1\;-\;\frac{\left(1-{\text{R}}^{2})\times (n-1\right)}{\left(n-1-k\right)}$$

where *N* is the sample size in the analysis, and *k* is the number of independent variables in the analysis.

## Results

**Figure 2.**
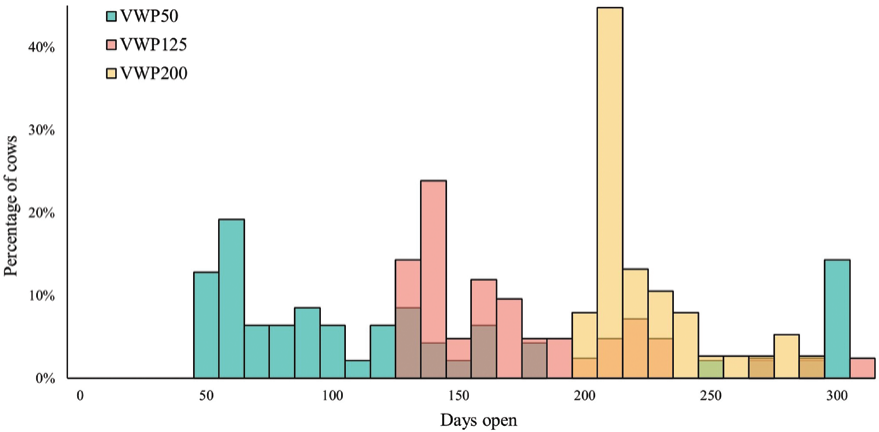
Distribution of days open of cows with a VWP of 50, 125, or 200 d (VWP50, VWP125, VWP200; *N* = 127).

From the 154 cows that entered the experiment, 127 cows conceived and had a second calf in the experiment. As a result of the VWP treatment, the number of days open was different for the three VWP groups (Figure 2). For cows in VWP50, mean days open was 107 ± 8 d, mean gestation length was 277 ± 0.8 d, mean dry period length was 41 ± 1 d, and mean CInt was 384 ± 9 d. For cows in VWP125, mean days open was 173 ± 7 d, mean gestation length was 278 ± 0.7 d, mean dry period length was 42 ± 1 d, and mean CInt was 452 ± 7 d. For cows in VWP200, mean days open was 224 ± 4 d, mean gestation length was 277 ± 1 d, mean dry period length was 43 ± 2 d, and mean CInt was 501 ± 4 d.

### Effect of voluntary waiting period during different phases of the lactation

#### First 305 d after calving 1

**Figure 3.**
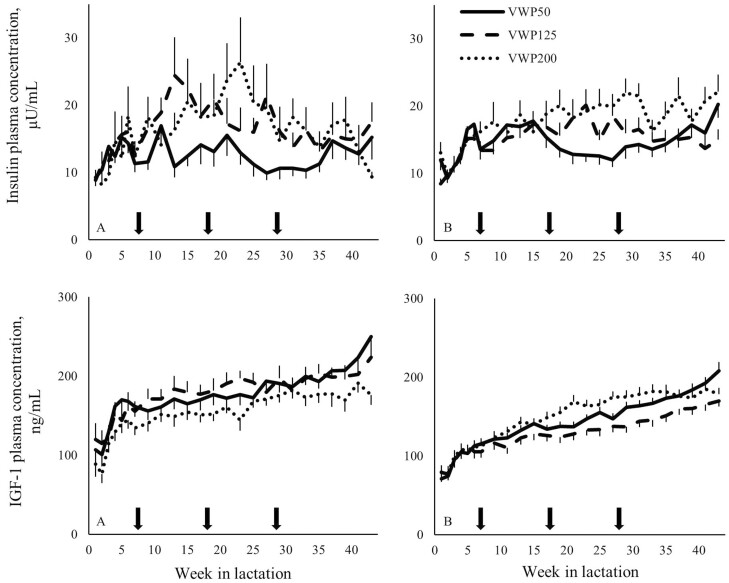
Plasma insulin concentration (µU/mL) and plasma IGF-1 concentration (ng/mL) for the first 305 d after calving 1 of primiparous (A) and multiparous (B) cows with a VWP of 50, 125, or 200 d (VWP50, VWP125, VWP200). Arrows depict the week in lactation from which insemination started for the respective VWP groups. Values represent mean ± SEM. Error bars are depicted either below or above the graphs.

Multiparous cows in VWP200 had a greater plasma insulin concentration compared with MP cows in VWP50 (*P* < 0.01) or VWP125 (*P* < 0.01) during the first 305 d after calving 1 (Table 1). Multiparous cows in VWP200 had a greater plasma IGF-1 concentration (*P* < 0.01), and a lower FPCM (*P* = 0.02) compared with MP cows in VWP125. Moreover, the effect of VWP on IGF-1 depended on lactation week and the effect of VWP on insulin tended to depend on lactation week (Figure 3).

**Table 1.** Plasma insulin and IGF-1 concentration, body condition, fat-and-protein-corrected milk, and energy partitioning for different periods in the lactation of primiparous and multiparous cows with a VWP after calving until first insemination of 50, 125, or 200 d (VWP50, VWP125, VWP200; LSM ± maximum SEM or CI)

	Primiparous cows			SEM (CI)	Multiparous cows			SEM (CI)	*P*-value					
	VWP50	VWP125	VWP200		VWP50	VWP125	VWP200		VWP	P^1^	W^2^	VWPxP	VWPxW	PxW
First 305 d after calving 1– n cows	14	15	12		39	34	39							
Insulin, µU/mL^3^	11.0^b^	13.4^a^	12.6^a,b^	(10.1–14.6)	12.3^b^	12.5^b^	14.5^a^	(11.7–15.3)	<0.01	0.05	<0.01	<0.01	0.07	0.41
IGF-1, ng/mL^4^	174.1	174.2	151.5	7.2	138.9^a,b^	126.4^b^	145.5^a^	4.3	0.34	<0.01	<0.01	<0.01	0.02	0.51
BCS (first 11 mo)	2.6	2.6	2.4	0.08	2.3	2.3	2.4	0.05	0.38	<0.01	0.08	0.19	0.35	0.13
BW, kg	593	578	592	15	700	692	687	9	0.60	<0.01	<0.01	0.73	0.57	0.50
Δ BW, kg/wk^5^	2.4	1.8	1.9	0.4	1.3	0.8	1.1	0.2	0.19	<0.01	<0.01	0.81	0.46	0.03
FPCM, kg/d^6^	26.6	27.7	28.0	1.1	34.3^a,b^	34.8^a^	32.3^b^	0.6	0.39	<0.01	<0.01	0.09	<0.01	<0.01
8 wk around end VWP – n cows	14	15	12		39	34	37							
Insulin, µU/mL^3^	12.6	16.5	14.3	(10.4–19.8)	13.4	13.7	17.6	(11.9––19.8)	0.04	0.68	0.42	0.05	0.70	0.81
IGF-1, ng/mL^4^	163.3	182.0	174.5	11.7	115.2^b^	126.4^b^	174.5^a^	7.0	<0.01	<0.01	0.28	<0.01	0.54	0.80
BCS (2 mo around end VWP)	2.7	2.6	2.4	0.13	2.4	2.3	2.5	0.08	0.83	0.04	0.37	0.19	0.91	0.74
Body weight, kg	561	571	595	16	683	685	695	9.5	0.17	<0.01	0.16	0.67	0.04	0.03
Δ BW, kg/wk^5^	2.2	2.1	1.7	1.1	−1.3	0.014	0.72	0.70	0.67	<0.01	0.58	0.35	0.05	0.68
FPCM, kg/d^6^	29.9	28.0	28.0	1.4	42.4^a^	36.9^b^	30.1^c^	0.8	<0.01	<0.01	0.01	<0.01	<0.01	0.13
Pregnancy period – n cows	13	14	9		34	28	29							
Insulin, µU/mL^3^	11.9^b^	15.4^a^	13.6^a,b^	(10.8–17.0)	13.9^c^	15.8^b^	18.5^a^	(13.0–19.7)	<0.01	<0.01	<0.01	<0.01	0.42	0.52
IGF-1, ng/mL^4^	210.8	221.7^†^	191.5^†^	9.3	175.3^b^	178.2^b^	198.5^a^	5.3	0.56	<0.01	<0.01	<0.01	<0.01	<0.01
BCS^7^	2.6	2.7	2.5	0.13	2.4^b^	2.6^b^	3.0^a^	0.07	0.04	0.38	<0.01	<0.01	<0.01	<0.01
BW, kg^7^	620	630	648	17	709	722	747	9.6	0.04	<0.01	<0.01	0.91	0.33	0.96
ΔBW, kg/wk^5^^,^^7^	2.9	3.4	2.7	0.5	2.5^b^	2.9^a,b^	3.6^a^	0.2	0.20	0.93	<0.01	0.07	0.72	0.31
FPCM, kg/d^6^^,^^7^	26.8	25.7	26.9	1.5	30.0^a^	26.6^b^	22.6^c^	0.8	<0.01	0.92	<0.01	<0.01	0.02	<0.01

^1^P = parity (primiparous or multiparous) in the first lactation in the experiment.

^2^W = week in lactation; month for BCS.

^3^Transformed data are back transformed; and confidence interval is shown.

^4^Insulin-like growth factor 1.

^5^ΔBW = ΔBody weight: difference between mean BW in 1 wk and mean BW in the next week.

^6^Fat-and-protein-corrected milk.

^7^Analyzed in the final 34 wk before dry-off, only cows with dry period (*N* = 124).

^a,b,c^Different superscript indicates a difference among LSM within the row and within the parity (*P* < 0.05).

^†^Similar symbol indicates a trend in difference among LSM within the row and within the parity (*P* < 0.10).

Primiparous cows in VWP125 had a greater plasma insulin concentration compared with PP cows in VWP50 (*P* = 0.01) during the first 305 d after calving 1. During the first 305 d after calving 1, the VWP did not affect the plasma IGF-1 concentration, or the FPCM of PP cows. The VWP did not affect the BCS, BW, or ΔBW.

#### Eight week around the end of the VWP.

**Figure 4.**
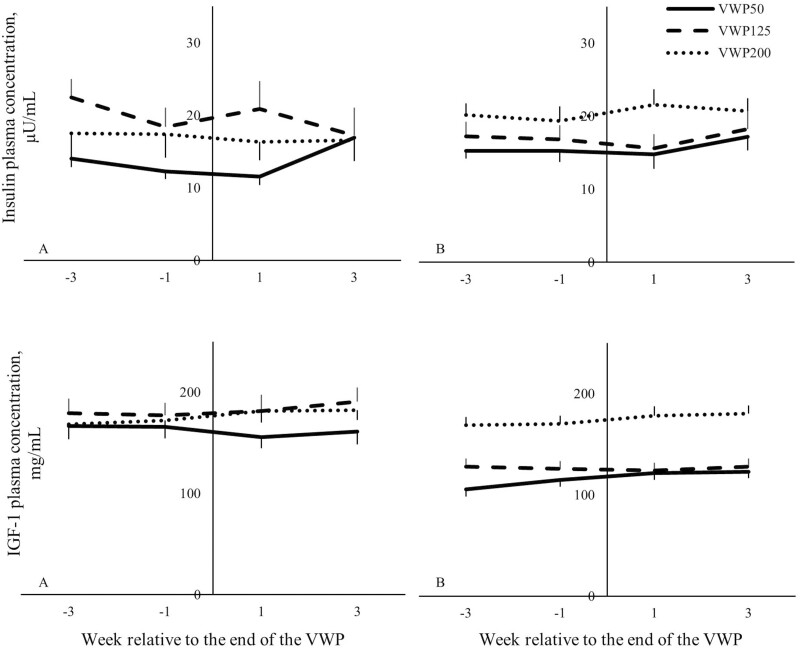
Plasma insulin concentration (µU/mL) and plasma IGF-1 concentration (ng/mL) for the 8 wk around the end of the VWP of primiparous (A) and multiparous (B) cows with a VWP of 50, 125, or 200 d (VWP50, VWP125, VWP200). Values represent mean ± SEM. Error bars are depicted either below or above the graphs.

Multiparous cows in VWP200 had a greater plasma insulin and IGF-1 concentration compared with MP cows in VWP125 (*P* < 0.05) or VWP50 (*P* < 0.01) during the 8 wk around the end of the VWP (i.e., around the start of the insemination period; Figure 4). For MP cows, the FPCM was lowest in VWP200, intermediate in VWP125, and greatest in VWP50 (*P* < 0.01 for all comparisons).

For PP cows, the VWP did not affect the plasma insulin and IGF-1 concentration, or the FPCM during the 8 wk around the end of the VWP. The BW or ΔBW did not differ among the three VWP in the different weeks or months around the end of the VWP. Moreover, the VWP did not affect the BCS.

#### *Pregnancy period*.

For MP cows, the plasma insulin concentration was greatest in VWP200, intermediate in VWP125, and lowest in VWP50 (*P* ≤ 0.02 for all comparisons). Multiparous cows in VWP200 had a greater plasma IGF-1 concentration compared with MP cows in VWP125 (*P* = 0.04) or VWP50 (*P* < 0.01).

**Figure 5.**
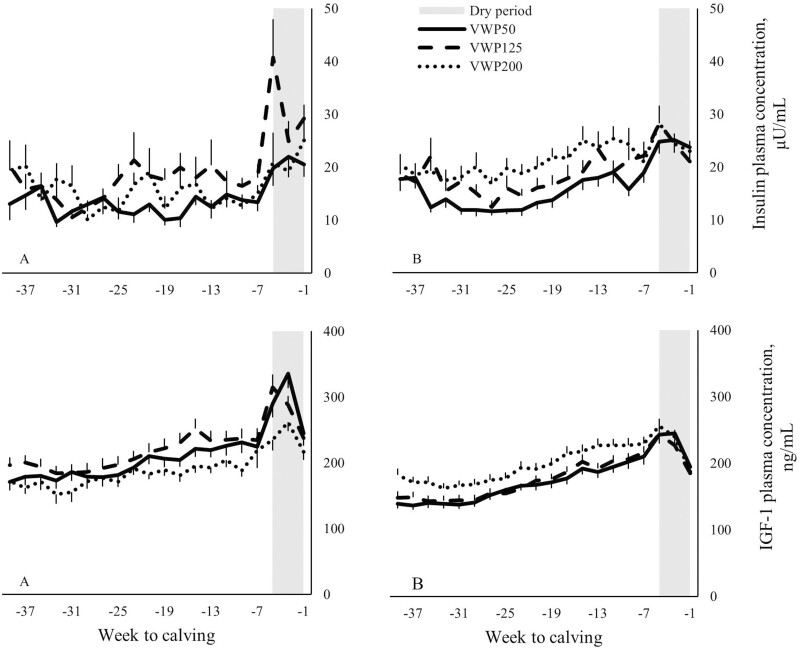
Plasma insulin concentration (µU/mL) and plasma IGF-1 concentration (ng/mL) for the pregnancy period of primiparous (A) and multiparous (B) cows with a VWP of 50, 125, or 200 d (VWP50, VWP125, VWP200). The dry period of the cows is illustrated in gray. Values represent mean ± SEM. Error bars are depicted either below or above the graphs.

Primiparous cows in VWP125 had a greater plasma insulin concentration compared with PP cows in VWP50 (*P* < 0.01) during the pregnancy period (Figure 5). Primiparous cows in VWP125 tended to have a greater plasma IGF-1 concentration compared with PP cows in VWP200, and the plasma IGF-1 concentration of PP cows in VWP50 did not differ from the other 2 VWP.

During the final 34 wk before dry-off (40−6 wk dry period), cows in VWP200 had a greater BW (697 kg) compared with cows in VWP50 (665 kg; *P* = 0.03). Multiparous cows in VWP200 had greater BCS compared with MP cows in VWP125 or VWP50 (*P* < 0.01) and gained more BW per week compared with MP cows in VWP50 (*P* < 0.01). During these final 34 wk before dry-off, for MP cows, the FPCM was lowest in VWP200, intermediate in VWP125, and greatest in VWP50 (*P* ≤ 0.01 for all comparisons). During the final 34 wk before dry-off, the VWP did not affect the BCS or ΔBW of PP cows.

### Effect of voluntary waiting period on metabolic status around calving 2

**Figure 6.**
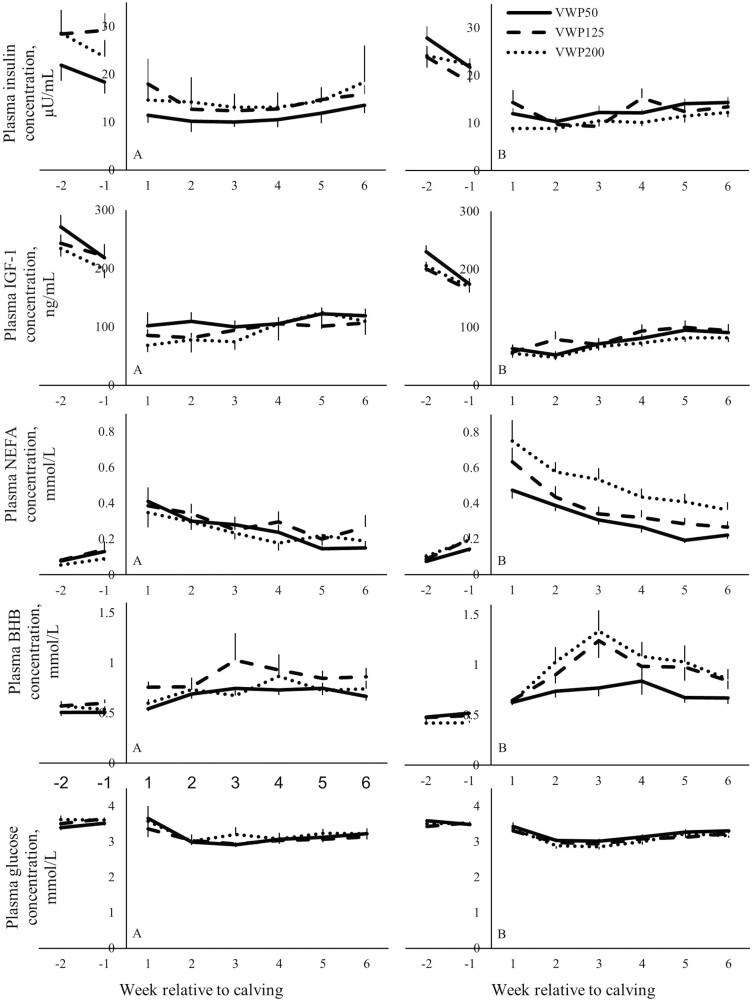
Plasma insulin (µU/mL), IGF-1 (ng/mL), NEFA (mmol/L), BHB (mmol/L), and glucose (mmol/L) concentration from 2 wk before until 6 wk after calving 2 of primiparous (A) and multiparous (B) cows with a VWP of 50, 125, or 200 d (VWP50, VWP125, VWP200). Parity (primiparous or multiparous cows) refers to the parity of the cow during the first lactation in the experiment. Values represent mean ± SEM. Error bars are depicted either below or above the graphs.

Multiparous cows in VWP200 had a lower plasma BHB concentration compared with MP cows in VWP50 (*P* = 0.02) during the 2 wk before calving 2 (Table 2). The VWP did not affect the plasma BHB concentration of PP cows before calving 2. During the 2 wk before calving 2, the VWP did not affect the BW, DMI, the total DMI (15.8 kg/d ± 0.17), the ratio PMR: concentrate (8.8 ± 0.36) or the EB. Moreover, the VWP did not affect the plasma NEFA, glucose, insulin, or IGF-1 concentration during the 2 wk before this calving (Figure 6).

**Table 2.** Body condition, DMI of concentrate and PMR, EB and plasma metabolites and hormones from 2 wk before until 6 wk after calving 2 of primiparous and multiparous cows with a VWP after calving until first insemination of 50, 125, or 200 d (VWP50, VWP125, VWP200; LSM±SEM or CI)

	Primiparous cows			SEM (CI)	Multiparous cows			SEM (CI)	*P*-value					
	VWP50	VWP125	VWP200		VWP50	VWP125	VWP200		VWP	P^1^	W^2^	VWPxP	VWPxW	PxW
*N* cows	13	14	9		34	28	29							
Week −2 to −1														
BW, kg	718	708	749	42	768	773	752	21	0.94	0.08	0.36	0.56	0.66	0.96
DMI concentrate, kg/d	1.8	1.7	1.7	0.07	1.7	1.7	1.6	0.04	0.27	0.11	<0.01	0.57	0.88	0.96
DMI PMR, kg/d^3^	14.0	13.9	14.8	0.81	14.6	13.7	13.6	0.44	0.57	0.60	<0.01	0.35	0.76	0.05
EB, kJ/BW^0.75^^4^	246	250	261	47	232	221	206	26	0.98	0.23	0.26	0.82	0.61	0.9999
NEFA, mmol/L^5^	0.09	0.09	0.06	(0.04–0.12)	0.09	0.11	0.11	(0.07–0.14)	0.51	0.05	<0.01	0.20	0.81	0.20
BHB, mmol/L^5^	0.50	0.57	0.55	(0.44–0.65)	0.49^a^	0.46^a,b^	0.41^b^	(0.38–0.52)	0.33	<0.01	0.60	0.03	0.41	0.42
Glucose, mmol/L^5^	3.46	3.57	3.61	(3.36–3.74)	3.53	3.48	3.51	(3.41–3.60)	0.41	0.31	0.18	0.12	0.08	0.18
Insulin, µU/mL^5^	18.5	26.3	22.2	(14.5–33.3)	21.9	18.7	20.9	(15.9–25.3)	0.60	0.39	<0.01	0.05	0.56	0.51
IGF-1, ng/mL	248.2	240.2	215.0	18.4	199.3	185.2	191.9	10.5	0.34	<0.01	<0.01	0.50	0.18	0.56
Week 1 t 6														
BCS	2.5	2.9	2.8	0.2	2.7^b^	3.0^b^	3.5^a^	0.1	<0.01	0.01	0.03	0.08	0.58	0.96
BW, kg	637	630	647	20	702	694	725	12	0.30	<0.01	0.09	0.89	0.98	0.96
DMI concentrate, kg/d^5^	8.0	8.0	7.8	(7.4–8.2)	7.9	7.8	7.8	(7.6–8.0)	0.34	0.18	<0.01	0.44	0.39	<0.01
DMI PMR, kg/d^3^	15.1	14.3	14.8	0.7	15.8	14.4	13.9	0.4	0.05	0.97	<0.01	0.37	0.20	<0.01
EB, kJ/BW^0.75^^4^	−202	−188	−132	51	−203	−213	−296	31	0.93	0.046	<0.01	0.08	<0.01	0.61
NEFA, mmol/liter^5^	0.21	0.23	0.19	(0.14–0.29)	0.26^b^	0.30^b^	0.41^a^	(0.22–0.48)	0.21	<0.01	<0.01	0.01	0.19	0.98
BHB, mmol/L^5^	0.65	0.80	0.69	(0.55–0.93)	0.66	0.81	0.84	(0.60–0.93)	<0.01	0.21	<0.01	0.37	0.26	0.21
Glucose, mmol/liter^5^	3.14	3.07	3.18	(2.91–3.39)	3.16	3.07	3.04	(2.93–3.27)	0.51	0.54	<0.01	0.51	0.89	0.76
Insulin, µU/mL^5^	10.0	12.2	11.4	(8.4–14.4)	11.1	10.3	9.3	(8.3–12.3)	0.53	0.17	<0.01	0.08	0.32	0.25
IGF-1, ng/mL	109.3	97.6	90.8	12.2	75.8	77.5	67.7	7.3	0.36	<0.01	<0.01	0.71	0.37	0.65

^1^P = parity (primiparous or multiparous) in the first lactation in the experiment.

^2^W = week relative to calving; month for BCS.

^3^PMR = partially mixed ration, consisting of grass silage, corn silage, soybean meal, and wheat meal.

^4^EB = energy balance, calculated as the difference between intake of NE and requirements of NE for maintenance, milk production, and pregnancy.

^5^Transformed data are back transformed, and confidence interval is shown.

^a,b^Different superscript indicates a difference among LSM within the row and within the parity (*P* < 0.05).

During the 6 wk after calving 2, for MP cows, BCS was greatest in VWP200, intermediate in VWP125, and lowest in VWP50. Multiparous cows in VWP200 had greater plasma NEFA concentration compared with MP cows in VWP50 (*P* < 0.01) or VWP125 (*P* = 0.04). In week 1 after calving 2, cows in VWP200 had a more negative EB compared with cows in VWP50 (−302 vs. −160 kJ/BW^0.75^, *P* = 0.02). Moreover, cows in VWP125 had a greater plasma BHB concentration (0.80 mmol/liter) compared with cows in VWP50 (0.65 mmol/liter, *P* < 0.01), and the plasma BHB concentration of cows in VWP200 (0.76 mmol/liter) did not differ from the other two VWP after calving 2.

The VWP did not affect the BCS or plasma NEFA concentration of PP cows. Cows in VWP50 had a greater total DMI (21.7 kg/d) compared with cows in VWP200 (20.3 kg/d, *P* = 0.04) and tended to have a greater total DMI compared with cows in VWP125 (20.5 kg/d, *P* = 0.09). The BW, DMI of PMR or concentrate, the ratio PMR: concentrate (2.9 ± 0.052), or the plasma glucose, insulin or IGF-1 concentration were not different among the three VWP during the first 6 wk after calving 2.

### Individual cow characteristics

First, the effects of VWP, parity, and their interaction on the three variables: mean FPCM in the final 6 wk before dry-off, mean BCS in the final 12 wk before dry-off, and FPCM per day of CInt and on the 23 cow characteristics were tested (Table 3). Parity affected all three variables, as well as part of the cow characteristics in the first 6 weeks of lactation (EB, DMI, plasma glucose concentration), all cow characteristics between calving and pregnancy, and part of the characteristics in the final week before successful insemination (milk production, lactose content, BW, SCC, and plasma IGF-1 concentration). The VWP affected all three variables, and part of the cow characteristics, mainly in the final week before pregnancy.

**Table 3.** FPCM end lactation, BCS end lactation, FPCM/d CInt, and cow characteristics between calving and successful insemination of cows with a VWP after calving until first insemination of 50, 125, or 200 d (VWP50, VWP125, VWP200) that completed the first lactation in the experiment and had a dry period (*N* = 124; LSM±SEM or CI)

	VWP			SEM (CI)	*P*-value		
	VWP50	VWP125	VWP200		VWP	P^1^	VWPxP
FPCM end lactation, kg/d	22.5^a^	19.4^b^	18.2^b^	1.0	<0.01	<0.01	ns
BCS end lactation	2.6^a^	2.9^b^	3.2^b^	0.11	<0.01	<0.01	0.01
FPCM/ dCInt, kg/d	27.4^†^	26.7	25.2^†^	0.73	0.06	<0.01	ns
Cow characteristics							
First 6 wk							
EB, kJ/BW^0.75^	−210	−203	−191	24.6	0.83	<0.01	ns
DMI concentrate, kg/d	5.6	5.6	5.5	0.03	0.08	<0.01	ns
DMI PMR, kg/d	12.1	12.0	11.7	0.29	0.62	<0.01	ns
NEFA, mmol/liter^2^	0.24	0.27	0.26	(0.20–0.31)	0.47	0.37	ns
BHB, mmol/liter^2^	0.69	0.74	0.69	(0.63–0.80)	0.45	0.66	ns
Glucose, mmol/liter^2^	3.35	3.30	3.38	(3.21–3.48)	0.57	<0.01	ns
Between calving and pregnancy							
Peak milk production, kg milk/d	39.6	38.9	38.1	1.1	0.58	<0.01	ns
Day of peak milk production	48	55	52	3.5	0.3	<0.01	ns
Slope to peak, kg milk/d^2^^,^^3^	0.25	0.20	0.23	(0.16–0.29)	0.17	<0.01	ns
Slope peak – pregnancy, kg milk/d^2^	−0.12^b^	−0.07^a^	−0.06^a^	(0.05–0.14)	<0.01	<0.01	<0.01
Slope final 3 wk to pregnancy, kg milk/d	−0.05	−0.07	−0.09	0.02	0.21	<0.01	ns
Final week before pregnancy							
Average milk production, kg/d	33.6^a^	29.4^b^	26.9^b^	1.3	<0.01	<0.01	<0.01
Fat, %	3.86^b^	4.25^a^	4.51^a^	0.12	<0.01	0.79	ns
Protein, %	3.49^b^^†^	3.63^a,b,^^†^	3.76^a^	0.05	<0.01	0.28	ns
Lactose, %	4.60^a^	4.55^a,b^	4.49^b^	0.03	0.01	<0.01	0.099
Fat: protein ratio	1.11^b^	1.17^a,b^	1.20^a^	0.03	0.02	0.3	ns
BW, kg	627^†^	639	653^†^	9.2	0.09	<0.01	ns
BCS^4^	2.4	2.4	2.6	0.10	0.18	0.18	ns
SCC (× 1000)^2^	55^†^	76	93^†^	(39–134)	0.08	<0.01	ns
Insulin, µU/mL^2^	12.8	16.2	14.8	(10.6–19.8)	0.21	0.6	ns
IGF-1, ng/mL	153.9	169.4	172.8	9.4	0.22	<0.01	0.02
Prior data							
Previous 305-d production, kg^5^	8,189	8,231	8,147	251	0.97	<0.01	ns
Breeding value persistency	102.4	103.2	102.1	0.7	0.5	0.03	ns

^1^P = parity (primiparous or multiparous) in the first lactation in the experiment.

^2^Transformed data are back transformed; and confidence interval is shown.

^3^Slope from day 10 in lactation until day of maximum production.

^4^Average in the final month before pregnancy.

^5^Previous (multiparous) or expected (primiparous) 305 d milk production.

^a,b^Different superscript letters indicate a difference among LSM within the column within one variable (*P* < 0.05).

^†^Similar symbol indicates a trend in difference among LSM within the row and within the parity (*P* < 0.10).

**Figure 7.**
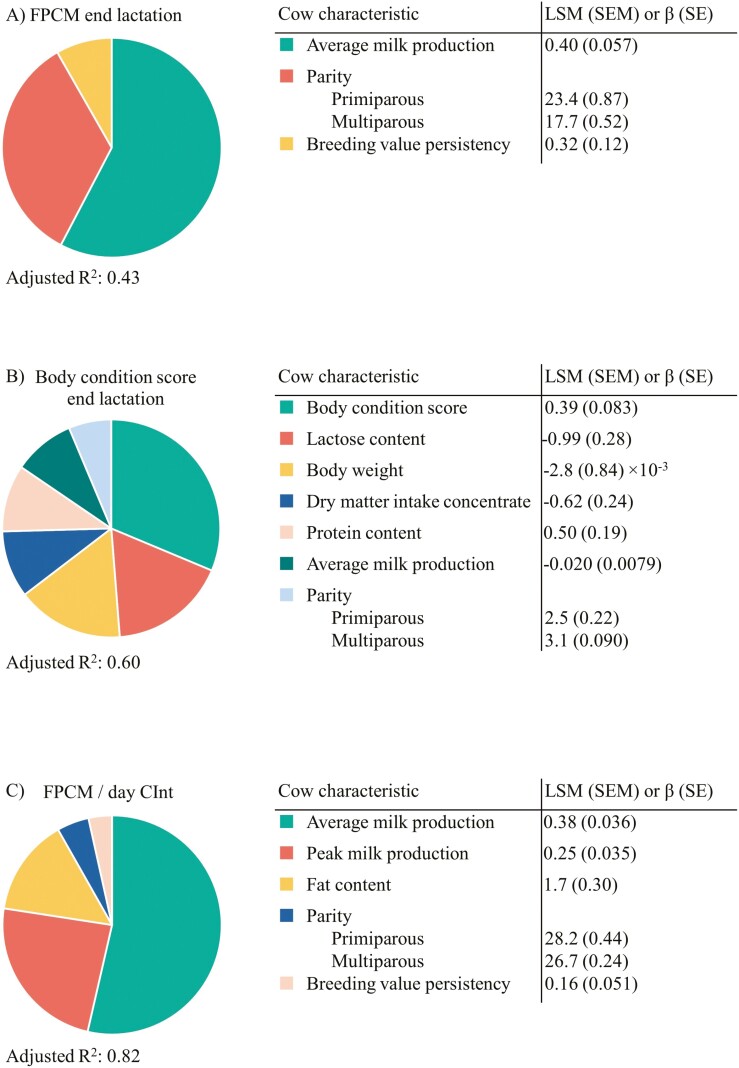
Cow characteristics that remain in the multivariable model to predict FPCM end lactation, BCS end lactation, and FPCM per day of CInt of cows with a VWP of 50, 125, or 200 d. Parity always remained in the model. Size of slices are based on type 3 sums of squares.


For the prediction of mean FPCM in the final 6 wk before dry-off, nine cow characteristics were selected at *P* < 0.20 from the univariate analyses (Supplementary Table S1). These were included in the first multivariable model, next to parity and their interactions with parity. After backward selection, the final multivariable model included three cow characteristics (Figure 7A). With this model, the FPCM end lactation was predicted as follows:

$${\text{FPCM}}_{end\;lac}=-27.2+\text{Par + 0.40 }\times \;{\text{Milk}}^{\text{Average}}+\;0.32\;\times \text{ BVpers}$$

where Par is the parity effect (PP: Par = 5.6, MP: Par = 0), Milk^AVERAGE^ is the average milk production in the final week before successful insemination, and BVpers is the breeding value for persistency. The residuals in this model were on average 3.5 kg FPCM/d. The adjusted R^2^ of the final multivariable model was 0.43, and the adjusted R^2^ of the model with VWP and parity (i.e., the model used in Table 3) was 0.19.

For the prediction of mean BCS in the final 12 wk before dry-off, 13 cow characteristics were selected at *P* < 0.20 from the univariate analyses. These were included in the first multivariable model, next to parity and their interactions with parity. After backward selection, this final multivariable model included seven cow characteristics (Figure 7B). With this model, the BCS production end lactation was predicted as follows:


$${\text{BCS}}_{end\;lac}=-7.2+\text{Par + 0.020 }\times \;{\text{Milk}}^{\text{Average}}+\;0.50\;\times \text{ Prot}-0.99\;\times \text{ Lac}+0.0028\;\times \text{ BW}+0.39\;\times \text{ BCS}-0.62\times \text{DMIcon}$$

where Par is the parity effect (PP: Par = −0.6, MP: Par = 0), Milk^AVERAGE^ is the average milk production in the final week before successful insemination, Prot is the protein content in the final week before successful insemination, Lac is the lactose content in the final week before successful insemination, BW is the average BW in the final week before successful insemination, BCS is the BCS in the final month before successful insemination, and DMIcon is the DMI of concentrate in the first 6 wk of lactation. The residuals in this model were on average 0.3. The adjusted R^2^ of the final multivariable model was 0.60, and the adjusted R^2^ of the model with VWP, parity, and their interaction (i.e., the model used in Table 3) was 0.32.

For the prediction of FPCM per day of CInt, 17 cow characteristics were selected at *P* < 0.20 from the univariate analyses. These were included in the first multivariable model, next to parity and their interactions with parity. After backward selection, this final multivariable model included five cow characteristics (Figure 7C). With this model, the FPCM per day of CInt was predicted as follows:


$$\frac{\text{FPCM}}{\text{Day CInt}}=18.9+\text{Par}+0.25\times {\text{Milk}}^{\text{peak}}+1.6\times \text{Fat}+0.38\times {\text{Milk}}^{\text{Average}}+0.16\times \text{BVpers}$$

where Par is the parity effect (PP: Par = 1.5, MP: Par = 0), Milk^PEAK^ is the peak milk production between calving and successful insemination, Milk^AVERAGE^ is the average milk production in the final week before successful insemination, Fat is the fat content in the final week before successful insemination, and BVpers is the breeding value for persistency. The residuals in this model were on average 1.4 kg FPCM/d. The adjusted R^2^ of the final multivariable model was 0.82, and the adjusted R^2^ of the model with VWP and parity (i.e., the model used in Table 3) was 0.15.

## Discussion

This study aimed to investigate how extending the VWP from 50 until 125 or 200 d affected metabolic status, ­hormones, and body condition development throughout the lactation and the start of the next lactation. When the VWP is extended, insemination takes place later in lactation. At this moment, daily milk yield can be expected to have decreased (Gaillard et al., 2016). For MP cows in the current study, indeed FPCM decreased around the end of the VWP when the VWP was increased. Possibly, lower milk production around the end of an extended VWP can explain the improved fertility, as reported earlier for the current experiment (Ma et al., 2022a) and in an earlier study (Niozas et al., 2019a). In the current experiment, cows in VWP200 had more normal ovarian cycles (18 to 24 d in length) around the end of the VWP and fewer days until pregnancy after the end of the VWP (Ma et al., 2022a). Also, earlier studies related a lower milk yield to fewer days open after the end of the VWP and greater first service conception rates (Wathes et al., 2007; Niozas et al., 2019a). In the current study, MP cows in VWP200 had a greater plasma insulin and IGF-1 concentration around the end of their VWP compared with cows with a shorter VWP, which may also be related to the improved fertility (Fouladi-Nashta et al., 2007; Wathes et al., 2007; Garnsworthy et al., 2009). Around successful insemination, the plasma insulin concentration of MP cows did not differ among the three VWP groups, in contrast with the period around the end of the VWP. One explanation could be that the success of ­insemination depended on the concentration of insulin, as it did in an earlier study (Garnsworthy et al., 2009).

A delayed insemination when the VWP is extended also implies that pregnancy takes place later in lactation. During these first 305 d of lactation in the current study, MP cows in VWP200 had a greater plasma insulin concentration compared with MP cows in VWP50 or VWP125, and a greater plasma IGF-1 concentration compared with MP cows in VWP125, possibly related to the differences in time of pregnancy. In addition, MP cows in VWP200 produced 2.5 kg/d less FPCM compared with cows in VWP125 during this period. Already in the first 6 wk, MP cows in VWP200 produced numerically 2.2 kg/d less FPCM compared with cows in VWP125 (Burgers et al., 2021a). When culled and nonpregnant cows were excluded from the analysis, however, FPCM did not differ among the VWP groups in the first 305 d after calving 1. This could indicate that some cows that were diseased or nonfertile or both and later culled lowered the average FPCM in VWP200.

As pregnancy took place later in lactation for cows with an extended VWP, the pregnancy period was at a different time in lactation for the different VWP groups. During the pregnancy period, MP cows in VWP200 had a greater plasma insulin and IGF-1 concentration, greater BCS, greater BW gain, and lowest FPCM. Also, MP cows with a VWP of 125 d had a greater plasma insulin concentration and a lower FPCM compared with MP cows in VWP50 but did not differ from VWP50 in terms of body condition or IGF-1 concentration during pregnancy. This indicates that this more limited extension of the VWP compared with 200 d also resulted in more limited differences in body condition and metabolism during the pregnancy period. Primiparous cows in VWP125 had a greater plasma insulin concentration compared with PP cows in VWP50. Earlier, the IGF-1 and insulin concentration were greater at later lactation stages compared with earlier lactation stages (Gross et al., 2011). Also, in an earlier study, cows between 301 and 600 DIM had elevated plasma concentrations of IGF-1, leptin, and glucose, a decreased milk yield, and an increased BW compared with cows between 0 and 300 DIM, indicating more partitioning of energy toward BW later in lactation (Marett et al., 2011).

The BCS and BW gain before calving could affect the cow in the period around the next calving (Roche and Berry, 2006). Indeed, in the current study, MP cows with a VWP of 200 d had a greater BCS, a more negative EB, and a greater plasma NEFA concentration after the next calving. This may be related to their greater BW gain and BCS in the end of the previous lactation (Gärtner et al., 2019). Moreover, a more negative EB and greater NEFA concentration in early lactation of these cows could imply that an extended VWP of 200 d in the previous lactation may increase the risk for diseases related with EB and metabolic status, such as ketosis and laminitis Ingvartsen et al., 2003; Friggens et al., 2004). A VWP of 125 d did not affect metabolism or body condition after the next calving, indicating that an extension of the VWP until 125 d does not increase the risk for a compromised metabolism after the next calving. In the current study, ration of cows in the three VWP groups was the same, and all cows were fed the same PMR with concentrates separate in a concentrate feeder. Even though concentrate level was adjusted to milk yield after 100 d in lactation, the basal ration already supplied for approximately 22 kg of milk per day, which could have contributed to the increased BCS at the end of the lactation of cows with an extended lactation. In practice, farmers may reduce the amount of concentrate in the basal ration to limit the risk of body fattening at the end of the lactation.

The effect of an extended VWP was different for MP cows and PP cows. We did not find an effect of VWP on body condition or FPCM of PP cows. Moreover, in contrast to MP cows, for PP cows the VWP had no effect on body condition, EB, or metabolites after the next calving. Possibly, this is related to the lack of effect of VWP on milk yield at the end of the previous lactation in PP cows (Burgers et al., 2021a) which could have prevented fattening at the end of the lactation. The lack of effect that we reported for PP cows may be a true lack of effect but may also be related with a limited number of PP cows. This study does give a numerical indication for the effect of an extended VWP for PP cows, and it seems that primiparous cows can handle an extended lactation well. Also, in other studies on extended lactations, PP cows maintained their production longer than MP cows (Rehn et al., 2000; Lehmann et al., 2016). When PP cows have an extended lactation, however, it takes more time before they become a, usually more productive, MP cow. In contrast, if the number of lactations stays the same, extending the lactation of PP cows may be beneficial for the lifetime production of a cow.

In the current study, a greater milk production before successful insemination and a greater breeding value for persistency were related with a greater FPCM end lactation. At the same time, these cows have a greater chance for a high milk production at the end of their lactation when applying a short VWP and with that they might have an increased risk for mastitis during the dry period (Rajala-Schultz et al., 2005). Other studies also reported that some cows could be better able to maintain their milk production in extended lactations compared with other cows, depending on individual characteristics (Lehmann et al., 2017; Kok et al., 2018). Farmers already used specific cow characteristics to select their cows for an extended VWP, such as parity, milk production level, peak production, and body condition (Burgers et al., 2021b).

A greater BCS, BW, and protein content before successful insemination was related with an increased BCS end lactation. Earlier, a greater nadir BCS after calving was positively related with protein content in the first 60 d of lactation (Roche et al., 2007). As an extended VWP increases the risk for an increased BCS end lactation (Niozas et al., 2019b; Burgers et al., 2021a), cows with higher BCS, BW, and protein content before insemination might be less suitable for an extended VWP. A greater milk production and lactose content before insemination was related with a reduced BCS end lactation, and as such cows with a greater milk production and lactose content before insemination could be more suitable for an extended VWP. In the current study, the lactose content before insemination was negatively correlated to the log-transformed SCC before insemination (r = −0.56, *P* < 0.01), which may explain the relationship with BCS at the end of the lactation. Possibly, cows with a greater SCC have a reduced milk production, and therefore more risk for fattening at the end of the lactation.

In our study, glucose in the first 6 wk of lactation and insulin and IGF-1 in the final week before successful insemination did not remain in the multivariate models after backward selection. Other studies, however, indicated that cows with lower concentrations of insulin and IGF-1 partition more energy reserves toward milk production than toward body reserves, resulting in greater lactation persistency (Kolver et al., 2007; Kay et al., 2009; Delany et al., 2010). This could imply that cows with a lower concentration of insulin, IGF-1, and glucose in late lactation or maybe already in early lactation could be more suitable for extended lactations. Possibly, the relationships between metabolites, hormones, energy partitioning, BW, BCS, milk production, milk content, and SCC prevented all these factors together to be selected by the model in our study, so that only the most profound factors remained, in this case mainly milk production and BCS.

The prediction models in this study use information that is available until the successful insemination of a cow. This is based on the approach of farmers in our network that already apply extended lactations on their farms (Burgers et al., 2021b). Most of these farmers evaluate the progress of the lactation of a cow and use the most recent information of milk production or body condition to decide whether to inseminate a cow in estrous, or to wait until the next estrous. Another approach could be to evaluate a cow in early lactation and decide on a cow specific VWP in the first part of the lactation of the individual cow (Supplementary Figure S1). The predictability of the model using information in early lactation was reduced compared with the model using information while lactation progresses, which might be related to both the amount of information available as well as the timing of the data collection closer to the timing of insemination and end of the lactation.

In practice, when farmers are interested in extending the VWP for part of their herd, they can use the prediction models in this study to select cows in their herd with a reduced risk for low milk production or fattening at the end of the lactation for an extended VWP. On other farms or with other cows, different cow characteristics could be relevant to predict the FPCM or the BCS at the end of the lactation, or the FPCM per day of CInt. Moreover, in commercial dairy practice, definition of a successful extended lactation is subject to a farmer’s individual approach and could also include reproductive performance, specific milk components, or simply a reduction in frequency of calving moments or a reduction in number of surplus calves. In addition, in the current study, the measurements are not done at the same time of the year for cows in different treatments. As things on a farm often change over time, this may have affected the results, while in practice, farm conditions like feed availability or feed quality might contribute to the decision of the farmer to extend the lactation of an individual cow or not. Part of the cow characteristics that we found, such as milk yield before insemination, peak production, and body condition, are already used by farmers for the selection of cows for an extended VWP (Burgers et al., 2021b). This could indicate that the prediction models created in this experiment may point the way for selecting individual cows for an extended VWP in practice.

## Conclusion

For PP cows, the VWP did not affect milk production or metabolism during their lactation and start of the next lactation, indicating PP cows can handle an extended lactation very well. For MP cows, a VWP of 200 d resulted in less milk and more BW gain during pregnancy. Moreover, these cows had a more severe negative EB during the start of the next lactation. Variation among cows, especially in parity, milk production and BCS, could call for an individual approach for extending the VWP. Especially for MP cows, an individually customized VWP can prevent fattening end lactation and associated problems in the start of the next lactation, while still having the beneficial effects of a lower frequency of calvings.

## Supplementary Material

skad194_suppl_Supplementary_Appendix

skad194_suppl_Supplementary_Figure_1A

## Abbreviations:

BCS: body condition score
BHB: β-hydroxybutyrate
BW: body weight
CInt: calving interval
DIM: days in milk
DMI: dry matter intake
EB: energy balance
FPCM: fat- and protein-corrected milk
IGF-1: insulin-like growth factor 1
LSM: least-squares mean
MP: multiparous
NEFA: nonesterified fatty acids
PMR: partially mixed ration
PP: primiparous
SCC: somatic cell count
VWP: voluntary waiting period
ΔBW: difference in body weight between two subsequent week
