# Supplementary material for: Effect of voluntary waiting period on metabolism of dairy cows during different phases of the lactation
Source: J Anim Sci. 2023 Jun 9;101:skad194. doi: 10.1093/jas/skad194 (PMC10351575; doi:10.1093/jas/skad194)
Supplement: skad194_suppl_Supplementary_Appendix [file skad194_suppl_supplementary_appendix.docx]

**APPENDIX**

**Table A1.** The effect (*P*-value) of cow characteristics between calving and successful insemination on 1. FPCM^1^ in the final 6 wk before dry-off, 2. BCS in the final 12 wk before dry-off, and 3. FPCM per day of CInt^2^, of cows with a voluntary waiting period after calving until first insemination of 50, 125, or 200 days (VWP50, VWP125, VWP200) that completed the first lactation in the experiment and had a dry period (n = 124). All models included parity next to the tested cow characteristic.

|  | 1. FPCM end | 2. BCS end | 3. FPCM / day CInt |
| --- | --- | --- | --- |
| First 6 wk |  |  |  |
| Energy balance, kJ/BW^0.75^ | 0.09 | 0.58 | <0.01 |
| Dry matter intake concentrate, kg/d | 0.56 | <0.01 | 0.199 |
| Dry matter intake partially mixed ration, kg/d | 0.38 | 0.82 | <0.01 |
| NEFA, mmol/L^3,4^ | 0.40 | 0.60 | <0.01 |
| BHB, mmol/L^3,4^ | 0.07 | 0.18 | <0.01 |
| Glucose, mmol/L^4^ | 0.45 | 0.26 | <0.01 |
| Between calving and pregnancy |  |  |  |
| Peak production, kg milk/d | 0.01 | 0.49 | <0.01 |
| Day of peak production | 0.72 | 0.09 | 0.87 |
| Slope to peak, kg milk/d^4,5^ | 0.26 | 0.67 | <0.01 |
| Slope peak – pregnancy, kg milk/d^4^ | 0.46 | 0.54 | 0.25 |
| Slope final 3 wk to pregnancy, kg milk/d | 0.69 | 0.57 | 0.58 |
| Final week before pregnancy |  |  |  |
| Milk production, kg/d | <0.01 | <0.01 | <0.01 |
| Fat, % | 0.31 | <0.01 | 0.12 |
| Protein, % | <0.01 | <0.01 | <0.01 |
| Lactose, % | <0.01 | <0.01 | 0.1 |
| Fat : protein ratio | 0.52 | 0.04 | 0.88 |
| Body weight, kg | 0.49 | <0.01 | <0.01 |
| Body condition score^6^ | 0.05 | <0.01 | <0.01 |
| Somatic cell count^4^ | 0.22 | 0.01 | 0.28 |
| Insulin, µU/mL^4^ | 0.60 | 0.84 | 0.30 |
| IGF-1, ng/mL^3^ | <0.01 | <0.01 | <0.01 |
| Prior data |  |  |  |
| Previous 305-d production, kg^7^ | 0.31 | 0.85 | <0.01 |
| Breeding value persistency | <0.01 | 0.03 | 0.01 |

^1^ FPCM = fat-and-protein-corrected milk.

^2^ CInt = calving interval.

^3^ NEFA = non-esterified fatty acids; BHB = β-hydroxybutyrate; IGF-1 = insulin-like growth factor 1.

^4^ Non-normal data were transformed data to approximate a normal distribution.

^5^ Slope from day 10 in lactation until day of maximum production.

^6^ Average in the final month before pregnancy.

^7^ Previous (multiparous) or expected (primiparous) 305-d milk production.

**Figure A1.**

Cow characteristics that remain in the multivariable model to predict FPCM end lactation, BCS end lactation, and FPCM per day of CInt of cows with a voluntary waiting period of 50, 125, or 200 d, using only cow characteristics available in the first 6 wk after the first calving in the experiment: milk production in the previous lactation (MP cows) or expected milk production (PP cows), the breeding value for persistency, EB, DMI of concentrate and PMR, plasma NEFA, BHB, glucose, insulin, and IGF-1 concentration, milk production, fat, protein, and lactose content, fat to protein ratio, BW, BCS, and SCC. Parity always remained in the model. Size of slices are based on type 3 sums of squares.
