# Supplementary figures and images for: Effect of voluntary waiting period on metabolism of dairy cows during different phases of the lactation
Source: J Anim Sci. 2023 Jun 9;101:skad194. doi: 10.1093/jas/skad194 (PMC10351575; doi:10.1093/jas/skad194)

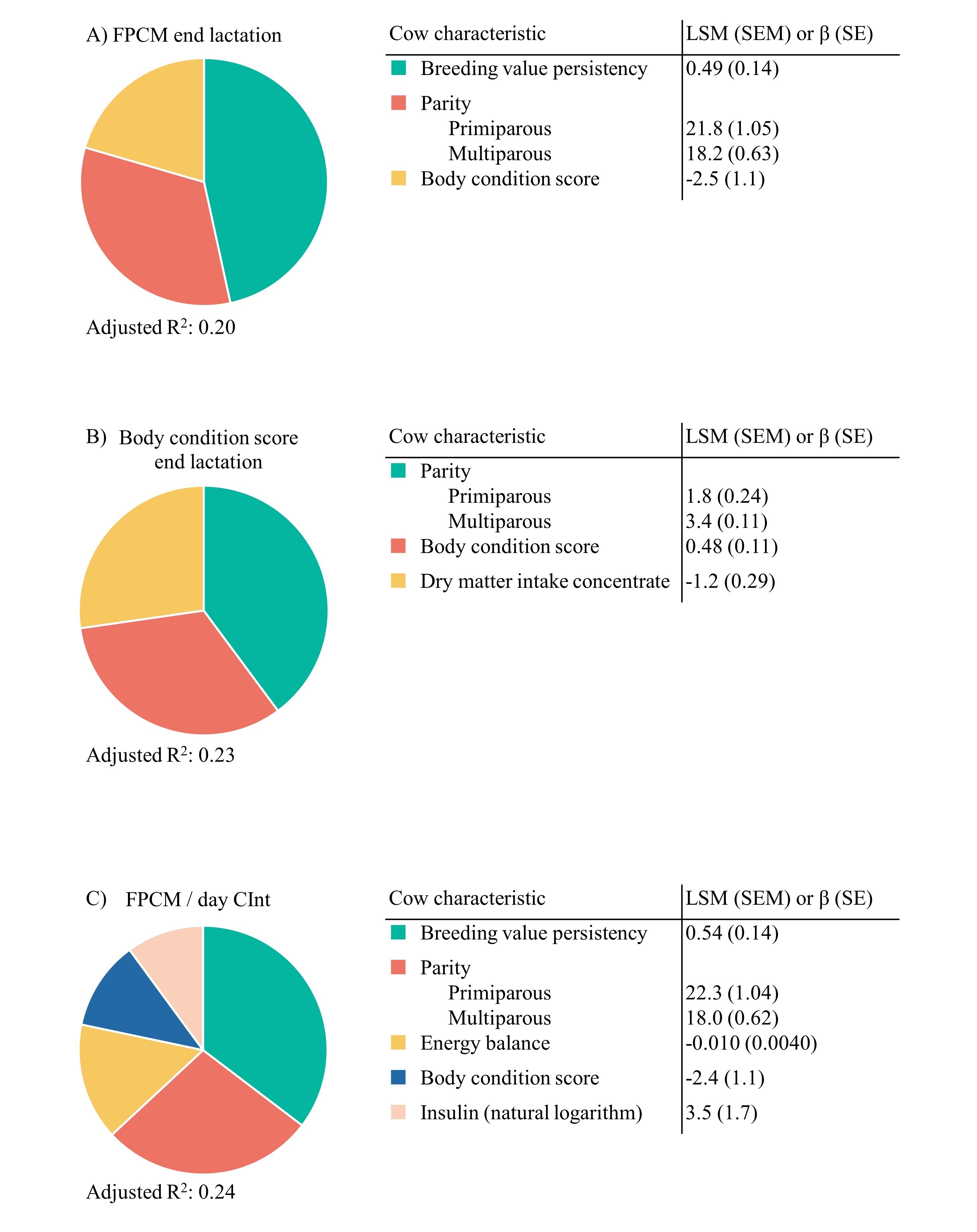

Supplement: skad194_suppl_Supplementary_Figure_1A [file skad194_suppl_supplementary_figure_1a.jpeg]
